# Conceptions of patient safety through the prism of social representations of intensive care nurses

**DOI:** 10.17533/udea.iee.v39n2e06

**Published:** 2021-06-15

**Authors:** Rejane Santos Barreto, Maria Lúcia Silva Servo, Alba Benemérita Alves Vilela, Elaine Guedes Fontoura, Sinara de Lima Souza, Neuranides Santana

**Affiliations:** 1 Nurse, Master´s degree. Residence in Intensive Care, Universidade Estadual de Feira de Santana Bahia, Brazil. Email: rejebarreto@gmail.com. Corresponding author. Universidade Estadual de Feira de Santana Universidade Estadual de Feira de Santana Bahia Brazil rejebarreto@gmail.com; 2 Nurse, Ph.D. Full Professor at Universidade Estadual de Feira de Santana, Brazil. Email: mlsservo@uefs.br Universidade Estadual de Feira de Santana Universidade Estadual de Feira de Santana Brazil mlsservo@uefs.br; 3 Nurse, Ph.D. Professor at Universidade Estadual do Sudoeste da Bahia, Brazil. Email: alba_vilela@hotmail.com Universidade Estadual do Sudoeste da Bahia Universidade Estadual do Sudoeste da Bahia Brazil alba_vilela@hotmail.com; 4 Nurse, Ph.D. Full Professor at Universidade Estadual de Feira de Santana, Brazil. Email: elaineguedesfont@uol.com.br Universidade Estadual de Feira de Santana Universidade Estadual de Feira de Santana Brazil elaineguedesfont@uol.com.br; 5 Nurse, Ph.D. Full Professor at Universidade Estadual de Feira de Santana, Brazil. Email: sinarals@uefs.br Universidade Estadual de Feira de Santana Universidade Estadual de Feira de Santana Brazil sinarals@uefs.br; 6 Nurse, Ph.D. Associate Professor at Universidade Federal da Bahia, Brazil. Email: neuranides@gmail.com Universidade Federal da Bahia Universidade Federal da Bahia Brazil neuranides@gmail.com

**Keywords:** patient safety, intensive care units, nurses., seguridad del paciente, unidades de cuidado intensivo, enfermeras y enfermeros, Segurança do paciente, unidades de terapia intensiva, enfermeiras y enfermeiros

## Abstract

**Objective.:**

To analyze the concepts of patient safety from the perspective of the social representations of intensive care nurses.

**Methods.:**

An exploratory, qualitative and quantitative study, based on the Theory of Social Representations, was conducted in a large hospital in northeastern Brazil, with 20 intensive care nurses. Data collection took place in 2019, using the techniques of free word association test and semi-structured interview. The lexicons apprehended in the test were processed by the *OpenEvoc* software, by prototypical analysis of the evocations, and for the interview data, thematic content analysis was used.

**Results.:**

In the composition of the central nucleus, the elements of surveillance, knowledge, identification, communication, and quality stood out, and in the constitution of the peripheral system of the social representations of intensive care nurses permeate care, attention, attitudes, and normative aspects. The triangulation of the findings outlined three thematic categories: Central dimensions of critical patient safety; Attitudinal dimensions for patient safety in intensive care; Normative dimensions linked to the safe handling of the patient in the ICU.

**Conclusion.:**

The social representations of intensive care nurses reveal that the critical patient's conceptions of security involve effective surveillance and communication, promotion of a safe environment based on risk prevention, use of guides and protocols, teamwork, and the sense of responsibility and commitment to individuality of being cared for, elements that for this social group, are the differential for assertive and safe care.

## Resumen

**Objetivo.:**

Analizar los conceptos de seguridad del paciente desde la perspectiva de las representaciones sociales de los enfermeros de cuidados intensivos.

**Métodos.:**

Estudio exploratorio, cualitativo y cuantitativo, basado en la Teoría de las Representaciones Sociales, realizado en un hospital del noreste de Brasil, con 20 enfermeras de cuidados intensivos. La recolección de datos se realizó en 2019, utilizando las técnicas de prueba de asociación de palabras libre y entrevista semiestructurada. Los léxicos aprehendidos en la prueba se procesaron ​​por el software *OpenEvoc*, mediante análisis prototípico de las evocaciones. Para los datos de la entrevista se utilizó análisis de contenido temático.

**Resultados.:**

En la composición del núcleo central se destacaron los elementos de vigilancia, conocimiento, identificación, comunicación y calidad. De otro lado, en la constitución del sistema periférico de las representaciones sociales de las enfermeras de la UCI permean los cuidados, la atención, las actitudes y los aspectos normativos. La triangulación de los hallazgos describió tres categorías temáticas: Dimensiones centrales de la seguridad del paciente crítico; Dimensiones actitudinales para la seguridad del paciente en cuidados intensivos; y Dimensiones normativas vinculadas al manejo seguro del paciente en UCI.

**Conclusión.:**

Las representaciones sociales de los enfermeros de UCI revelan que las concepciones de seguridad del paciente crítico implican una vigilancia y comunicación efectiva, la promoción de un ambiente seguro basado en la prevención de riesgos, el uso de guías y protocolos, el trabajo en equipo y el sentido de responsabilidad y compromiso con la individualidad del ser cuidado, elementos que para este grupo hacen la diferencia de un cuidado asertivo y seguro.

## Resumo

**Objetivo.:**

Analisar as concepções de segurança do paciente pelo prisma das representações sociais de enfermeiros intensivistas.

**Métodos.:**

Estudo exploratório, quali-quantitativo, fundamentado na Teoria das Representações Sociais, realizado em um hospital de grande porte do nordeste brasileiro, com 20 enfermeiros intensivistas. A coleta de dados ocorreu em 2019, sendo utilizadas as técnicas de teste de associação livre de palavras e entrevista semiestruturada. Os léxicos apreendidos no teste foram processados pelo software *OpenEvoc* por análise prototípica das evocações, e para os dados da entrevista, utilizou-se a análise de conteúdo temática.

**Resultados.:**

Na composição do núcleo central destacaram-se os elementos vigilância, conhecimento, identificação, comunicação e qualidade, e na constituição do sistema periférico das representações sociais de enfermeiros intensivistas permeiam cuidado, atenção, atitudes e aspectos normativos. A triangulação dos achados delineou três categorias temáticas: Dimensões centrais da segurança do paciente crítico; Dimensões atitudinais para segurança do paciente na terapia intensiva; Dimensões normativas atreladas ao manejo seguro do paciente na UTI.

**Conclusão.:**

As representações sociais de enfermeiros intensivistas revelam que as concepções de segurança do paciente crítico perpassam pela vigilância e comunicação efetiva, promoção de ambiente seguro tendo como base a prevenção de risco, uso de guias e protocolos, trabalho em equipe, e pelo sentido de responsabilidade e compromisso com a individualidade do ser cuidado, elementos que para este grupo social, são o diferencial para o cuidado assertivo e seguro.

## Introduction

Health care organizations and services have shown growing concern for patient safety, and this phenomenon has been widely discussed to ensure and ensure that the care provided proceeds efficiently, free from harm and practices.(1) In Brazil, the landmark of attention to patient safety was the implementation of the National Patient Safety Program on April 1, 2013, demarcating actions, protocols, and guidelines for safe practices to be adopted as premises fundamental in this area.(2) This movement for safe care is essential in all health settings; however, in the Intensive Care Units (ICU) where complex care is imperative, with a diversity of technologies necessary for the care of critical patients, the patient safety dimension becomes a multi-professional challenge, especially for nurses who are on the front line of care. The intensive care nurses are responsible for a large part of the care actions and are in a privileged position to reduce the possibility of an incident, detect complications early, and perform the necessary steps to minimize damage.(3) Thus, not only these professionals but the entire multidisciplinary team must have the skills to act assertively in favor of reliable practices and the quality of care provided.

In these scenarios, the influence of the clinical conditions of critically ill patients on adverse events is frequent, that is, occurrences with the potential to cause damage, given the patient's instability, the need for risky interventions, and invasive procedures. Thus, critical patients are particularly more vulnerable to risks and complications from non-assertive behaviors.(4) In this sense, considering the peculiar characteristics described in the ICU context, with anchoring to hard technologies, meeting a different patient severity profile of the other services, multiple demands of processes and activities, it is important to get closer to the new dimensions of knowledge about patient safety, such as those in the area of the social representation, considering that safety is a theme permeated by subjectivity and relationships established in the daily work that can trigger behaviors and attitudes that influence professional practice but are liable to reframing.(5) However, knowing the social imaginary, that is, the senses, meanings, knowledge, values, and knowledge socially elaborated by intensive care nurses, access their social representations, their ideas, concepts, concepts, understanding, and attitudes towards the phenomenon of patient safety.

Social representations are concepts of everyday life from collective interaction without restricting individual perceptions. They are equivalent in our society to myths, traditional belief systems, that is, to the contemporary version of common sense.(6) Given these considerations, we understand that patient safety investigations favor management decision-making and interventions, modify care practices and organizational culture. Therefore, we outline the following research objective: to analyze patient safety concepts from the perspective of the social representation of intensive care nurses.

## Methods

This is exploratory research with a qualitative and quantitative approach, based on the theoretical and methodological contribution of the Theory of Social Representations,(7) product of the master's thesis developed in 2019, in the Professional Master's Program in Nursing of the Health Department of the *Universidade Estadual de Feira de Santana*, Bahia (Brazil), entitled “Social Representations of Intensive Care Nurses on Patient Safety.”(8) To ensure ethical rigor, this research was developed only after approval by the Research Ethics Committee with human beings, Opinion 3,239,115/2019. Participation in the research was conditioned to the acceptance and signing of the Informed Consent Form by the participants after being aware of the theme, justification, objectives, benefits, and risks of the research, following the ethical precepts of research with human beings.(9,10) We adopted methodological rigor, following the guidelines of the qualitative research checklist, Consolidated Criteria For Reporting Qualitative Research (CORE-Q),(11) in all stages.

The research scenario was a large private hospital, located in the northeastern region of Brazil, which has a general and specialized ICU. The approach to the field occurred through the dissemination and presentation of the research project at the institution in a meeting with the entire clinical staff and individual formalization through a printed invitation delivered to each potential participant in the study. On the occasion, the interviewer approached the field and highlighted the possibilities of reaching research interventions for the studied reality. Twenty intensive care nurses participated in the research, defined by inclusion/exclusion criteria, refusal, and data saturation. We selected nurses who had a minimum of six months of service, who worked in general ICU and direct care for the patient, we excluded and coordinators for carrying out activities related to management, professionals who were on maternity/health leave or vacation during the data collection period. There was a refusal to participate and three nurses were not interviewed, due to the end of data saturation collection, which occurred when the essence of the testimonies did not point to new information, and those obtained ensured quantity, relevance, and consistency of content to meet the proposed objective of the research.

Data collection took place using the following techniques: Free Words Association Test (FWAT) and semi-structured interviews. FWAT, as a projective technique, helped to identify the content implicit in the construction of the object for the social context under study, allowing to obtain evidence of its representations.(12) The research participant was presented with the expression “patient safety”, who acted as an inductive term corresponding to the representation phenomenon that is being investigated, asking him to evoke five words or expressions and to subsequently perform the hierarchy of the evoked terms in increasing order of importance to assign values from the most important to the least important.

After the application of the FWAT, we carried out a semi-structured interview, seeking to deepen knowledge about the social and collective imaginary, that is, the reality experienced, and to access other latent elements of social representations. As a comprehensive collection technique, the interview privileged social interaction covered the subjective field of ideas and meanings through the individuals' spontaneity and interaction.(13) We collected the research data between June and August 2019, with the consent of the service, in a private room, with an average duration of 15 minutes. The collection instruments were previously tested and included objective data referring to the characterization of the participants and subjective questions that sought to meet the research objective. The contents of the FWAT and the interviews were recorded and transcribed *ipsis litteris*, returned to the participants for corrections, with no need for repetition and/or exclusion of content, and then submitted to analysis.

For the data collected at FWAT, a prototypical analysis of evocations was carried out using the OpenEvoc software,(14) which enabled the survey of the first social representations of what could be considered as central or peripheral elements, based on the hierarchy of Frequency and Average Order of Evocations (AOE) revealed in a table of four squares. This type of analysis has its validity based on the assessment of the salience of the representational elements quantitatively, when crossing Frequency and AOE, and the central elements of the representation are the most salient, being more present in the discourse. However, the salience is data that can also be found in peripheral elements.(15)

Considering the importance of multi-methodological analysis in studies of social representations, and aiming at a greater understanding, the empirical material produced by the interview followed the steps of thematic content analysis, allowing for an objective, systematic description of the manifest content of the communication.(16) In this process, three thematic groups were identified that outlined three analytical categories: Central dimensions of critical patient safety; Attitudinal dimensions for patient safety in intensive care; Normative dimensions linked to the safe handling of patients in the ICU, which reveal the concepts of patient safety through the prism of the social representations of intensive care nurses. We ensured the anonymity of the interviewees and the contents of the speeches were named by vasoactive drugs (gluconate, quelicin, nipride, ancoron, bicarbonate, diprivan, dobutamine, dopamine), considering the amount of occurrence of AE related to the drug cycle in the health scenarios.

## Results

In the composition of the researched group, the age of the 20 participants ranged from 28 to 53 years old, with a predominance of females. We observed a long variation of time of training/graduation in nursing (ranged between 4 and 30 years), length of experience in the ICU (ranged from 2 to 29 years), and the time of work at the institution researched (ranging from 10 months to 28 years). As for complementary training, except for one nurse, all had postgraduate degrees in the area of expertise, either by specialization or by a residency in intensive care. Most had a single work contract, as a dependent worker contract, with a workload of 44 hours per week.

The prototypical analysis of evocations based on the content learned in FWAT, in response to the expression “patient safety”, apprehended 100 lexicons evoked by intensive care nurses, 61 of these were different and 16 were identified by the OpenEvoc software as the most important and made part of the composition of the schematic representation. The construction of the table of four houses was performed through the calculation and combined analysis of the Average Order of Evocations (generated around 3.1) and the average frequency of words (generated around 4), as shown in Table 1.

**Table 1:** Social representations of 20 intensive care nurses on patient safety

Central Core Elements 1^st^ Quadrant	Central Core Elements 1^st^ Quadrant	Central Core Elements 1^st^ Quadrant	Elements of the Peripheral System (1^st^ periphery) 2^nd^ Quadrant	Elements of the Peripheral System (1^st^ periphery) 2^nd^ Quadrant	Elements of the Peripheral System (1^st^ periphery) 2^nd^ Quadrant
Lexicons	Frequency >= 4	AOE < 3.1	Lexicons	Frequency >= 4	AOE >= 3.1
Surveillance	6	2	Care	7	3.14
Knowledge	4	1	Attention	7	3.29
Identification	4	1.75			
Communication	4	2.5			
Quality	4	2.75			
Core Elements 3^rd^ Quadrant	Core Elements 3^rd^ Quadrant	Core Elements 3^rd^ Quadrant	Elements of the Peripheral System (2^nd^ periphery) 4^th^ Quadrant	Elements of the Peripheral System (2^nd^ periphery) 4^th^ Quadrant	Elements of the Peripheral System (2^nd^ periphery) 4^th^ Quadrant
Lexicons	Frequency < 4	AOE < 3.1	Lexicons	Frequency < 4	AOE >= 3.1
Responsibility	3	2	Team	2	3.5
Visibility	2	2	Planning	2	3.5
Commitment	2	2.5	Devices	2	4
Assistance	2	3	Individuality	2	4.5
Protocols	2	3			

* OME (Average Order of Evocations).

The processing established by the OpenEvoc software indicated that the set of words in the 1^st^ quadrant is the likely central nucleus, as they presented higher citation frequencies readily evoked, suggesting greater meaning for the researched social group and, consequently, characterize the ontological meaning of the representation. In that quadrant, the terms “vigilance and knowledge” are highlighted. The word “surveillance” was evoked 6 times (with AOE = 2), that is, the term was quoted twice in the first, second, and third positions in the order of the evocation rangmot, and indicates an attitudinal dimension towards patient safety. However, the lexicon "knowledge" has a greater degree of salience, that is, greater importance given by the group, since this term was promptly evoked every time in the first position (AOE = 1), denoting the elements of greater representativeness for the nurses. In this perspective, still composing the representations of the central nucleus, there are the terms "identification", "communication" and "quality", which occupied an equal position considering the frequency of evocations, although with differentiated AOE. The grouping of words from the central nucleus has associated characteristics and indicates a typical way of thinking of the researched group, constituting and reflecting the consensual basis that is shared collectively.

In the 2^nd^ quadrant of the schematic representation, there are the transition elements of the social representations that make up the first periphery. They represent the highest frequency of quotes later evoked and reinforce the ideas of the probable central nucleus. The terms "care" and "attention" seem to inductively refer to the essence of the intensive care nurse's work process, promoting an association between concrete reality and the central nucleus, based on the integration between what is lived collectively and individual experiences.

In the 3^rd^ quadrant there are the lexicons with the lowest frequency of citations, but readily evoked, which complement and discuss the central nucleus and the other peripheral elements and at the same time, represent tension in the object, as they may indicate changes or transitions in social representations. The terms "responsibility and commitment" denote attitudinal dimensions inherent in the work of assisting lives in situations of narrow limits between normality and abnormalities, associated with the complexity of the intensive care work process.

Returning to the table of four squares, we find that the lexicons "team", "planning", "devices" and "individuality", which make up the 4^th^ quadrant or second periphery, obtained the lowest frequencies of quotes evoked late. Therefore, they are the most distant elements of the central system and may suggest perceptions or individual experiences of the group regarding patient safety. From the representational structure presented in Table 1, safe intensive care is based on the dimensions: imagery (imaginary that sustain the representation), knowledge (refers to an acceptable basis for intensive care and the social cognitive self-reconstruction of this phenomenon in extreme situations), attitudinal (the attitudes that offer representation dynamics), and the normative (the pre-conceived constructions about representation, coming from common sense, but which are passive of reinterpretations).

The understanding of these dimensions and elements considered structuring was revealed by the contents of the speeches and outlined nuclei, categories, and thematic units:

### Thematic nucleus 1. Surveillance, communication, identification, attention, devices, knowledge, and quality

*Category*: *Central dimensions of critical patient safety*

*Thematic units: Security is the surveillance of people at the bedside, communication between shift changes, [...] attention to patient identification, attention to devices, the bed rail, fixations (Gluconate); It is to evaluate the individual not only with his illness but with everything around him, all with the knowledge that I think is paramount [...] (Quelicin); Safety is the pillar of our profession today, which will give quality to our service. (Bicarbonate).*

### Thematic nucleus 2. Care, visibility, responsibility, commitment, and individuality

*Category*: Attitudinal dimensions for patient safety in intensive care

*Thematic units:* *You try to make the most of that environment that is safe to provide your care, everything you can guarantee to prevent risks, risk of falling, risk of phlebitis, risk of various risks [...] (Nipride); I check everything, check if all risks and measures are prescribed for the patient (Ancoron); [...] it is the perception that will bring the commitment to individuality, the individuality that I say is the detail, that each patient is individual, there are different details of each one that will bring this to our safety (Bicarbonate).*

### Thematic nucleus 3. Assistance, planning, and protocols

*Category: Normative dimensions linked to the safe handling of patients in the ICU*

*Thematic units: [...] we have steps and work routines for almost all the procedures we do, and in case of doubt, you have the option to consult (Diprivan); [...] every time I feel insecure about some action I have to do, I go to the protocol (Dobutamine); Patient safety, it is part of the team's work process, how does the team do this work, [...] if there is a synchrony of communication between all teams (Dopamine).*

## Discussion

The category “central dimensions of critical patient safety” points out the collective imagination that supports the representation of the researched social group, involving perceptions attributed by intensive care nurses to the safety of clinically unstable individuals, based on continuous surveillance, effective communication, attention during intensive clinical and device management, that is, it reflects the conceptual basis of intensive care and, consequently, the quality of care offered.

In this sense, the element "surveillance", accessed from the inductive term "patient safety", represents the product of the activity of construction of reality, derived from information, values, attitudes, and impressions of daily practice.(6) Such a fact is justified by the observation that the ICU's main objective is to promote continuous and rigorous monitoring in the face of hemodynamic, ventilatory, fluid-electrolyte balance, management of vasopressor and sedative drugs, among other monitoring.(17) The representations learned to suggest that safe care should direct attention to the weaknesses in patient identification, prevention of falls and unintentional or iatrogenic exit of devices, elements of daily practice that can generate harm to patients and increase costs to organizations. The excerpt of the discourse that brings “knowledge as primordial” signals the importance of the scientific domain and the ability to evaluate the environment-disease-patient context as essential elements to guarantee safety.

Following the content of the speech and being part of the semantic universe of the central nucleus, the term “knowledge”, the act of perceiving or understanding through reason and or experience,(13) interacts with the element “care” of the first periphery, denoting connection between them. In this sense, nurses, when experiencing critical care, work with multitasking and with the automatism that the activities require. However, they need to exercise critical observation in the face of the situations experienced and use scientificity in directing their assistance.(3)

The apprehension of the category in the reflective analysis also reports patient safety to the quality dimension of the intensive nursing service. Similarly, the element “quality” was chosen at FAWT for the composition of the central nucleus, suggesting representativeness for the social group, despite the adversities of being a health field nuanced by hard technologies, an exhaustive and uninterrupted work process, risks inherent to procedures invasive, which can influence, compromise, or even distance the reach of excellence in daily work. However, the central dimensions presented reinforce the structure of nurses' social representations about assertive care, highlighting the meanings of professional practice and the daily socio-cognitive reconstructions of these professionals because of the understanding of patient safety, originated in the constructions, opinions, attitudes, values, feelings and collective experiences of the group.

The category “attitudinal dimensions for patient safety in intensive care”, originated from lexicons of the contrast zone and the peripheral system that offer dynamics to representations, that is, elements that reflect attitudes and actions for the safety of the critical patient's patient. The movement to build a safe environment for the provision of intensive care, portraying the work process and the flow of activities that are attentive to patient safety, was observed in the speeches that point to critical care based on patient knowledge and risk reduction. In this perspective, social representations signal the act of prevention as an effective barrier to avoid damage associated with health care and the potential risks that can be avoided through actions and attitudes aimed at complying with prescribed measures, verifications, and checking.

Patient safety is related to changes in the work process, that is, the way the human being produces and reproduces his existence, interfering in the way that the nurse performs his daily work.(17) Thus, the actions of conferences, checking described in the content of the discourse, if on the one hand demonstrate objectification, through the creation of superimposed barriers to minimize the risk of errors, exchange of patients and adverse events, on the other, they denote subjective implications, such as responsibility and commitment to the other. This subjective dimension reveals congruence with the constituent elements of the contrast zone of the representational structure, directs to a more critical look at the “patient safety” object, and reflects a positive safety culture because it signals ideas, meanings, feelings, from the lexicons: responsibility (duty, obligation), commitment (commitment, accountability), assistance (handling, surveillance, providing care).

The individuality was another meaning apprehended regarding the attitudinal dimension, observation of the particularities and singularities of the person being cared for. In this sense, we can infer that the assistance to the critical patient is marked by the application of greater knowledge by the nurse, who constantly directs his attention in the search for objective and subjective information from the client and objective data from the machinery. However, in the face of technological action, what is observed in daily practice is that care is based on the observations coming from the equipment to the detriment of individual observations.(18)

The complexity of the critical patient with its pluralities and demands, the hard technology used require the purposeful management of multiple resources and equipment. However, meeting the individualities and respecting the individual's diversity are shown to be inseparable mechanisms to care security.(4) It reflects the nurses' behavior, initiative, way of acting, that is, they portray attitudes, actions, daily work movements for patient safety.

The category “normative dimensions linked to the safe handling of patients in the ICU” reports preconceived constructions about the phenomenon under study, coming from common sense and representations linked to the work process. Similar to the content of the speeches, the “protocols” recognized by the researched group since the prototypical analysis, composing the contrast zone of the representational structure, were identified as devices/instruments that help to define, standardize and review the way of processing direct attention the health. Thus, they seem to direct the work, record the care performed, helping to solve or prevent a problem.(19)

These apprehensions reveal an organizational culture focused on safe care, because of the familiarity with which the intensivist nurses surveyed demonstrate by consulting the protocols in situations of doubt, insecurity, and the knowledge of this instrument as a risk management tool, as a guide for compliance with organized flows and work routines.

Teamwork and its interface with safety bring representations that suggest the alignment of communication between all peers responsible for assistance, being important for the assertive management of conducts. Although the lexicon "team" is considered an intermediate element in the structure of social representation based on FWAT due to its lower frequency of citation that is late evoked, denotes the perception by the group of multidisciplinary interdependence and the importance of group work for patient safety. Thus, it is necessary to perceive teamwork based on a transdisciplinary proposal that must be characterized by the intensity of exchanges between people and daily integrations, which are crystallized from intentionality and elements of communication.(20) However, for the product to be multidisciplinary integration, assertive communications must occur. In this sense, critical moments of threat to patient safety occur precisely due to the low integration of the parts and communication failures in interactions poorly established by the multi-professional team,(21) with effective communication being a challenge for critical care units.

Thus, the category “Normative dimensions linked to the safe handling of patients in the ICU” indicates that the focus on patient safety and the improvement of the quality of health care, in general, requires a systemic view to understand the process between professionals/organizations/sectors, compliance with routines and protocols to create an integrated, adequate and timely network to achieve the best results in a transdisciplinary way.

We concluded that the representations apprehended in the implicit and latent contents reveal that the concepts of patient safety include working in the search for actions, tools, methodologies, solutions, and strategies that aim to identify, make visible, prevent, reduce or mitigate risks, and then, minimize and/or eliminate the occurrence of AE and make complex care safe.

Also, despite the number of intensive care nurses interviewed, which can be considered a limitation of the study, the concepts presented by the researched group indicate a movement for assertive care. However, the observance and investment by health managers and multidisciplinary teams in daily practices that reflect the safe management of critical patients are still incipient. This highlights the potential of the result of this study, which may provoke new itineraries, reflections, training, and reframings regarding safety in these care scenarios.
